# EnsembleAge: enhancing epigenetic age assessment with a multi-clock framework

**DOI:** 10.1007/s11357-025-01808-1

**Published:** 2025-08-06

**Authors:** Amin Haghani, Ake T. Lu, Qi Yan, Juan Carlos Izpisua Belmonte, Pradeep Reddy, Victor Cheng, X. William Yang, Nan Wang, Khyobeni Mozhui, Kevin Murach, Alejandro Ocampo, Robert W. Williams, Mathias Jucker, Carina Bergmann, Jesse R. Poganik, Bohan Zhang, Vadim N. Gladyshev, Steve Horvath

**Affiliations:** 1 Department of Human Genetics, David Geffen School of Medicine, University of California Los Angeles, Los Angeles, CA USA; 2 Altos Labs, San Diego, CA USA; 3 Salk Institute for Biological Studies, La Jolla, CA USA; 4 Center for Neurobehavioral Genetics, Semel Institute for Neuroscience and Human Behavior, University of California Los Angeles, Los Angeles, CA USA; 5 Department of Psychiatry and Biobehavioral Sciences, David Geffen School of Medicine, University of California Los Angeles, Los Angeles, CA USA; 6 Department of Preventive Medicine, College of Medicine, University of Tennessee Health Science Center, Memphis, TN USA; 7 Department of Genetics, Genomics and Informatics, College of Medicine, University of Tennessee Health Science Center, Memphis, TN USA; 8 Exercise Science Research Center, Department of Health, Human Performance, and Recreation, University of Arkansas, Fayetteville, AR USA; 9 Cell and Molecular Biology Graduate Program, University of Arkansas, Fayetteville, AR USA; 10 Department of Biomedical Sciences, Faculty of Biology and Medicine, University of Lausanne, Lausanne, Switzerland; 11 EPITERNA SA, Epalinges, Switzerland; 12 Department of Genetics, Genomics and Informatics, University of Tennessee Health Science Center, College of Medicine, Memphis, TN USA; 13 Hertie Institute for Clinical Brain Research, University of Tübingen, Tübingen, Germany; 14 Division of Genetics, Department of Medicine, Brigham and Women’s Hospital, Harvard Medical School, Boston, MA USA; 15 Altos Labs, Cambridge, UK

**Keywords:** Epigenetic clocks, Biological age, MethylGauge dataset, EnsembleAge, DNA methylation, Aging biomarkers, Healthspan, Lifespan interventions, Mouse models, Stress response, Rejuvenation

## Abstract

Several widely used epigenetic clocks have been developed for mice and other species, but a persistent challenge remains: different mouse clocks often yield inconsistent results. To address this limitation in robustness, we present EnsembleAge, a suite of ensemble-based epigenetic clocks. Leveraging data from over 200 perturbation experiments across multiple tissues, EnsembleAge integrates predictions from multiple penalized models. Empirical evaluations demonstrate that EnsembleAge outperforms existing clocks in detecting both pro-aging and rejuvenating interventions. Furthermore, we introduce EnsembleAge HumanMouse, an extension that enables cross-species analyses, facilitating translational research between mouse models and human studies. Together, these advances underscore the potential of EnsembleAge as a robust tool for identifying and validating interventions that modulate biological aging.

**Supplementary Information:**

The online version contains supplementary material available at 10.1007/s11357-025-01808-1.

## Introduction

Epigenetic clocks have transformed aging research by providing molecular biomarkers that estimate biological age based on DNA methylation (DNAm) patterns [1–4]. Initially developed for humans, these clocks have since been adapted for rodent models, offering powerful tools to study aging and longevity interventions in preclinical settings. The first epigenetic aging clocks for mice were developed using Reduced Representation Bisulfite Sequencing (RRBS). Some of these clocks were tissue-specific, focusing on organs such as the liver [5] and blood [6]. However, subsequent efforts led to the development of multi-tissue clocks capable of capturing age-related DNAm changes across various organs throughout the lifespan [7–9]. These murine epigenetic clocks have demonstrated that longevity-enhancing interventions, including caloric restriction and genetic disruptions in growth hormone signaling resulting in dwarf mice are associated with decelerated epigenetic aging [5–8, 10, 11]. While RRBS-based methylation profiling provides broad genome coverage and cost-effectiveness, it also presents limitations. RRBS selectively enriches CpG-dense regions using the restriction enzyme MspI, which cuts at CCGG sites. In practice, only a subset of CpG sites is captured, leading to missing data in certain samples, particularly when sequencing depth is limited. This variability hinders the development of robust DNA methylation-based biomarkers that require consistent CpG site measurements across all samples. For example, if a CpG site critical for a biomarker is not measured in some samples, it reduces analytical reliability.

In contrast, methylation arrays, such as the Illumina Infinium platform, consistently measure the same set of CpG sites across all samples with high probe coverage and technical reproducibility, ensuring more reliable biomarker development. The Mammalian Methylation Array, a technology applicable across all mammalian species, addresses many of these limitations [12]. By targeting evolutionarily conserved CpGs, it enhances the likelihood that aging-related DNAm signatures in one species will translate to another [13, 14]. The array’s high precision stems from two key factors [12]: (1) the hybridization step selectively captures fully bisulfite-converted DNA strands, improving methylation signal detection, and (2) targeted CpG sites are assayed at high reproducibility, enhancing the reliability of age prediction models.

We and others have recently developed multiple epigenetic clocks for mice based on the Mammalian Methylation Array [12, 13, 15–17]. These clocks were constructed using elastic net regression to predict a log-linear transformation of chronological age. Other researchers have proposed alternative statistical approaches, including ridge regression and lasso regression [1], [8].

The numerous epigenetic clocks developed for mice and other species present significant challenges in standardization and comparability. Variability in clock selection across studies often results in inconsistencies in biological age estimation. For instance, if one study utilizes clock A while another relies on clock B, direct comparisons may be unreliable. One approach to addressing this reliability issue is the development of ensemble-based epigenetic clocks, which combine multiple individual clocks to enhance accuracy and consistency. By integrating predictions from multiple models, these ensemble clocks reduce biases inherent to any single clock and improve the robustness of biological age estimation.

A crucial prerequisite for constructing a biologically meaningful ensemble clock is the availability of standardized benchmarking datasets that incorporate DNAm measurements from validated pro-aging and rejuvenation interventions. To address this need, we leveraged MethylGauge, a comprehensive benchmarking dataset derived from 211 controlled perturbation experiments in mouse models. MethylGauge is a collection of data measured using the Mammalian Methylation Array, ensuring consistent measurement across experiments [12]. By aggregating DNAm data from multiple tissues and diverse longevity-related and stress-inducing interventions, MethylGauge provides a robust reference framework for evaluating the biological utility of individual epigenetic clocks.

Using MethylGauge, we systematically assessed the performance of multiple epigenetic clocks to identify those that maintain high predictive accuracy across various experimental conditions. We then developed EnsembleAge, an aggregated epigenetic clock designed to enhance accuracy and reduce false positives and false negatives when evaluating perturbation effects in mouse tissues. Unlike single-model clocks, EnsembleAge leverages multiple penalized regression methods. By standardizing epigenetic age estimation and enhancing reproducibility, the EnsembleAge clock facilitates robust evaluation of longevity interventions. While this study primarily focuses on mouse models, we also developed cross-species clocks applicable to both humans and mice (termed the HumanMouse version of the Ensemble clock). Establishing a reliable cross-species framework will be essential for advancing translational aging research.

## Results

### Development of the EnsembleAge clock

**Fig. 1:**
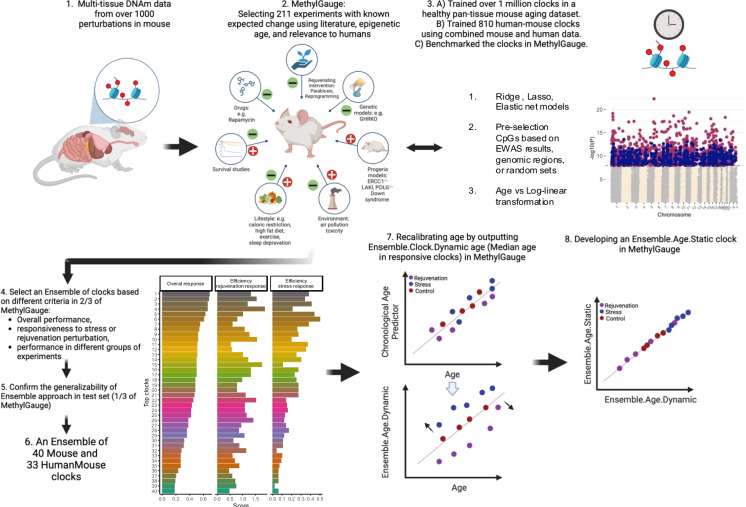
Schematic overview of the MethylGauge study design and EnsembleAge development. This figure outlines the key steps involved in constructing the MethylGauge dataset and developing the EnsembleAge clock. (1) More than 1,000 perturbation experiments were conducted in various mouse tissues, including liver, blood, brain, and others. (2) We compiled DNA methylation (DNAm) data into a comprehensive benchmark dataset called MethylGauge, encompassing 211 controlled perturbation models with documented phenotypic effects in mice. These models include a wide range of experimental conditions, many of which are directly related to longevity interventions—such as dietary modifications—and exposures to various stressors, including air pollution, genotoxic stress, and disease models like progeria and Huntington’s disease. (3) Model development: Clock training was limited to CpGs based on top EWAS results in different experiments. Three penalized regression models (ridge, lasso, and elastic net) were applied to the selected CpG sites. HumanMouse clocks are trained by combining mouse and human data and predicting the relative age (age/maximum species lifespan). HumanMouse clocks are limited to 2000 CpGs shared across all known BeadChip platforms (Human Infinium 450 K, EPIC(v1/v2) arrays and mammalian array). (4–6) Ensemble clock selection: The top performers of each group of studies are selected for EnsembleAge calculation. The generalizability of the model is tested in 1/3 of test experiments. (7) Chronological ages of the mouse data were calibrated by EnsembleAge.Dynamic (median prediction of predicted age in EnsembleAge clocks) to maximize the relationship to health outcome of an intervention. (8) An elastic net predictor of EnsembleAge is developed to also be applied via EnsembleAge.Static for quick screening of future data.

To address the limitations of current epigenetic clocks, particularly their lack of consistency, we developed the EnsembleAge clock. To generate distinct versions of individual clocks, we employed multiple construction methods, including three types of penalized regression models: ridge, lasso, and elastic net regression. The EnsembleAge clock leverages data from 211 controlled perturbation models in the MethylGauge dataset, which includes pro-aging (stressful) and rejuvenating interventions (Fig. 1). We analyzed methylation data from genetic disease models (e.g., Hutchinson Gilford progeria, Huntington’s disease), and rejuvenating interventions such as caloric restriction or transient cellular reprogramming based on the Yamanaka factors [13, 18]. This ensemble method allowed us to aggregate age predictions and calibrate the clock across various tissues and perturbations, producing a clock with greater sensitivity to both rejuvenation and stress responses (Fig. 1).

**Fig. 2:**
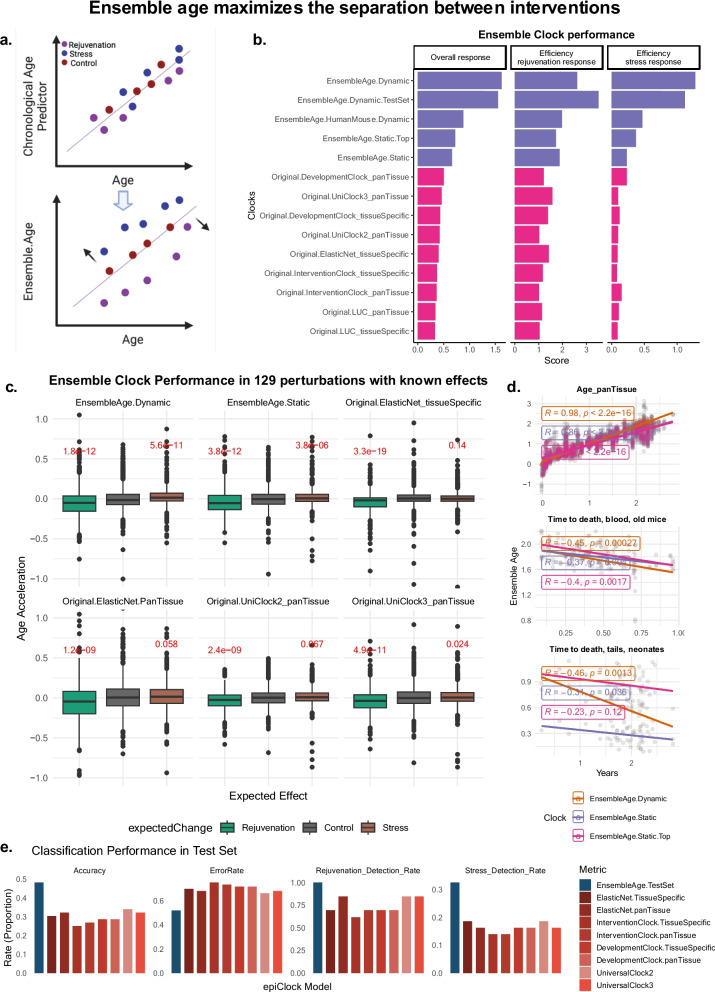
Performance of EnsembleAge clocks across MethylGauge (mouse perturbation benchmark dataset). **a **Schematic graph of Ensemble.Age, which recalibrates age to maximize the separation between interventions, rejuvenation, and stress responses. **b** Performance of Ensemble clocks vs the previously developed mouse clocks in MethylGauge: LUC clocks, developmental clocks, intervention clocks, elastic net clocks, and universal clocks. Clocks are ranked by their ability to respond in expected direction depending on stress or rejuvenation. The presented scores are ratios of significance in expected change weighted by significance of change. **c** Predicted age acceleration versus expected biological effects (e.g., stress, rejuvenation). The EnsembleAge.Dynamic and EnsembleAge.Static clocks demonstrate superior prediction accuracy for both stress and rejuvenation compared to other models. **d** Correlation between EnsembleAge clocks to chronological age (dynamic, *R* = 0.98; static, *R* = 0.86; Static.Top, *R* = 0.8), and time to death in adult mouse blood (dynamic, *R* = − 0.45; static, *R* = − 0.37; Static.Top, *R* = − 0.4) and neonate tail (dynamic, *R* = − 0.46; static, *R* = − 0.31; Static.Top, *R* = − 0.23). The original clocks are unable to detect a significant association for the time to death dataset (data not shown). **e** Classification performance of epigenetic clock models, highlighting accuracy, error rate, stress detection rate, and rejuvenation detection rate to evaluate their ability to distinguish stress-induced and rejuvenation-associated changes

The EnsembleAge clock produces two key outputs, both derived from calibrated age estimates in MethylGauge. The first, EnsembleAge.Dynamic, is the median predicted age across clocks that are most responsive to perturbations (|Z|> 2) within each dataset, ensuring an adaptive measure of epigenetic aging that maximizes its relationship to intervention outcomes. The term “dynamic” reflects that the clock composition changes with each test dataset, increasing sensitivity but risking overfitting. However, contrary to expectations, EnsembleAge.Dynamic demonstrated the highest sensitivity with lowest false positive and negative rates in test experiments (Fig. 2e).

The second output consists of static predictors of EnsembleAge.Dynamic, which are single elastic net models designed to predict recalibrated age (i.e., EnsembleAge.Dynamic or the most responsive clock age calibrated to intervention effects) instead of chronological age, maximizing intervention association. Unlike the dynamic version, static clocks are fixed in the training data and do not overfit. We developed two types of EnsembleAge.Static: one predicting the median EnsembleAge.Dynamic value, and another optimized for the most responsive clock (the clock with the highest Z-score in the expected direction). To enhance intervention responsiveness, we trained these static models exclusively on treated/perturbed animals. These single elastic net predictors, trained within MethylGauge, simplify the multi-clock approach while maintaining computational efficiency and broad applicability (Table S3).

Here are the steps for EnsembleAge development (Fig. 1).

1. *MethylGauge curation and normal aging dataset*: A total of 6224 DNAm samples from 211 tissue-specific strata across 44 lifespan intervention studies were curated for benchmarking based on literature review, relevance to epigenetic aging, and applicability to human aging studies. These experiments included dietary manipulations (e.g., caloric restrictions), stress exposures (e.g., high-fat diet, sleep disruption, air pollution), exercise, long lived genotypes (e.g., GHRKO, Snell Dwarf mice), partial reprogramming, parabiosis, various mouse progeria models (e.g., LAKI mice modeling Hutchinson–Gilford progeria). Each tissue-stratum contains age-matched control samples (C57BL/6 J mice) for direct comparison. In addition to MethylGauge, 1468 DNAm samples from 11 normal aging mouse tissues (blood, liver, heart, kidney, muscle, cerebral cortex, striatum, cerebellum, whole brain, fibroblast, skin, and tail) were used for clock training and aging signature identification.
2. *EWAS analysis:* Using the limma package in R, epigenome-wide association studies (EWAS) were conducted on different intervention datasets to identify CpGs associated with aging and perturbation effects. These EWAS results informed CpG pre-selection for clock development, with significant CpGs pre-filtered using an absolute z-score threshold of |z|> 2, where z was derived from the Fisher transformation of *p* values, ensuring biologically relevant and statistically robust selection. Beta values were analyzed to measure methylation levels at individual CpG sites.
3. *Epigenetic clock development*: To compare the performance of different epigenetic clocks in lifespan intervention experiments, we trained clocks using a dataset of 1468 DNAm samples from normal aging mice. Clock development was conducted using the “mlr” and “glmnet” packages in R. Penalized regression models (ridge, lasso, and elastic net) were applied to DNAm data to predict chronological age based on CpG methylation. The dataset was split into 70% training and 30% testing sets. Clocks were trained and optimized using tenfold cross-validation to minimize mean square error (MSE). Final models were trained on the full dataset after validation, and their performance was assessed in independent testing sets.
4. *Clock training and benchmarking*: We trained over 1 million clocks on a pan-tissue mouse aging dataset. All clocks were benchmarked within the MethylGauge dataset to assess their accuracy and responsiveness to interventions. The models were built using ridge, lasso, and elastic net regression techniques, with CpG sites pre-selected based on EWAS results, genomic regions, or random sets. To improve prediction accuracy, clocks incorporated various age transformation methods, including log-linear adjustments [15].
5. *Ensemble clock selection*: To assess the responsiveness of clocks to interventions, we developed multiple evaluation metrics. Since MethylGauge experiments have two expected outcomes—stress-induced aging acceleration and rejuvenation-induced deceleration—we applied a confusion matrix framework to quantify accuracy and error rates. Several metrics were used in this process, including the ratio of correct associations (based on expected direction), the median z-score of associations, and a z-score weighted ratio of correct responsiveness to stress or rejuvenation. These metrics were calculated across all experiments and within specific experimental groups. Based on these assessments, the top-performing clocks were selected in two-thirds of MethylGauge based on overall predictive performance, responsiveness to stress and rejuvenation perturbations, and consistency across different experimental categories. We did the clock benchmarking and selection in 2/3 of the MethylGauge. After benchmarking, a total of 40 mouse clocks were selected (Table S2).
6. *EnsembleAge.Dynamic calculation and generalizability testing*: Since these clocks were extensively benchmarked in MethylGauge, we expected higher sensitivity and specificity compared to conventional clocks. For each new experiment, we first identified the significant (responsive) clocks, then calculated the median z-score change across these clocks. The generalizability of this approach was tested using an independent test set (one-third of the remaining MethylGauge data) to evaluate its robustness and predictive accuracy. This validation confirmed that EnsembleAge.Dynamic consistently outperformed conventional epigenetic clocks in detecting intervention effects and capturing perturbation responses with high sensitivity and specificity.
7. *Calibration of age by EnsembleAge.Dynamic*: Age in MethylGauge was calibrated using two approaches: (1) *EnsembleAge.Dynamic*, defined as the median predicted age from responsive clocks (|Z|> 2), and (2) the predicted age from the most responsive EnsembleAge clocks (the clock with the maximum Z-score in the expected direction). These calibrations were designed to maximize the relationship between predicted age and intervention outcomes in mouse tissues. Only samples with unique EnsembleAge.Dynamic values were retained to ensure stability.
8. *Development of EnsembleAge.Static*: Two versions of *EnsembleAge.Static* were developed to predict the calibrated ages from the previous section. This approach collapses the multi-clock framework into a single, highly responsive clock for improved simplicity and reproducibility. An *elastic net predictor* was trained within MethylGauge to provide a computationally efficient, pre-calibrated alternative for future studies (Table S3).

### Human-mouse EnsembleAge clock development

The development of the human-mouse EnsembleAge clock followed the same methodology as the mouse-only version, using a merged human-mouse dataset to enable cross-species epigenetic age predictions. To achieve this, we combined methylation data from both species, restricting CpG selection to the 2,252 CpGs shared across the Mammal40k, EPIC, and 450k arrays for broad platform compatibility. The clocks were trained using ridge, lasso, and elastic net regression models, normalizing age by maximum species lifespan to account for differences in aging rates. The human dataset consisted of 81 blood samples (ages 53–92) labeled with GRIMAge2, which was used to enhance the clock’s relevance to human mortality risk. As with the mouse clocks, benchmarking and validation were performed within MethylGauge, ensuring high sensitivity and specificity in detecting biological age changes across species. This approach allowed for the creation of a robust Human-Mouse EnsembleAge clock capable of cross-species comparisons (Table S4).

### Assessment of EnsembleAge performance

The EnsembleAge clock demonstrated superior performance when compared to previously established clocks, such as the elastic [15], LUC [16], developmental, intervention, and universal clocks [13] (Fig. 2b). Across the MethylGauge dataset, the EnsembleAge clock significantly outperformed the other clocks in its ability to predict age acceleration in response to both stress and rejuvenation interventions (Fig. 2c). The dynamic and static versions of EnsembleAge showed robust correlation with chronological age (dynamic *R* = 0.98, static *R* = 0.86), as well as strong negative correlations with time to death in adult blood and neonate tail samples (Fig. 2d). In comparison, original published clocks failed to detect age acceleration or health outcomes in these datasets.

The EnsembleAge clock’s ability to separate stress and rejuvenation responses was particularly evident in stress-induced perturbations, where other models either underperformed or showed reverse relationships (Fig. 2c). These findings suggest that EnsembleAge is more attuned to detecting subtle health-related changes compared to existing clocks. Additionally, the HumanMouse version of this clock demonstrated excellent generalizability across different species (Fig. 2b), further supporting its use as a cross-species tool for biological age prediction [12].

### Performance of EnsembleAge across individual studies

**Fig. 3:**
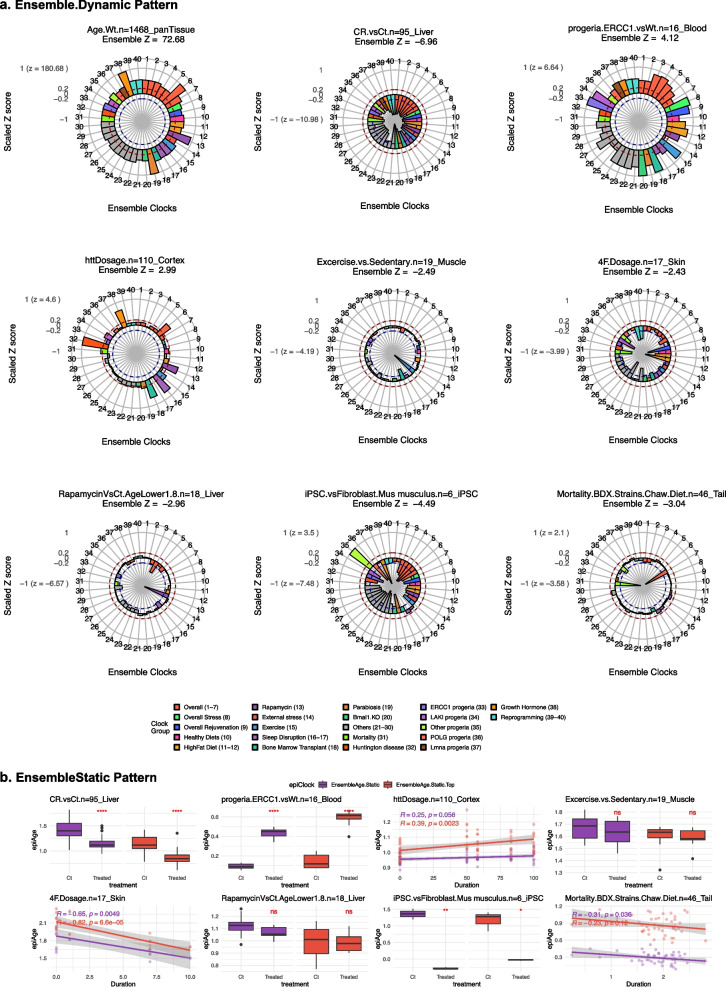
Performance of Ensemble clocks across strong and weak signal perturbations. Performance of Ensemble clocks across strong and weak signal perturbations. **a** Radar plot showing the response of 40 Ensemble clocks across different experiments. Scaled Z-scores (derived from *p* value transformation) were grouped by the best-performing clocks for each condition. Z > 2 was linearly scaled to [0.2, 1], − 2 ≤ Z ≤ 2 was mapped to [− 0.2, 0.2], and Z < −2 was scaled to [− 1, − 0.2]. This transformation preserves the directionality of effects while normalizing extreme values for comparability across conditions. Max Z scores are indicated in parentheses. Ensemble Z is the median of significant clocks. These radar plots improve the interpretability of clock responses across perturbations, showing the superior performance of EnsembleAge clocks. **b** Performance of Ensemble.Clock.Static in these experiments

To visualize the clock’s sensitivity across different perturbations, we used radar plots (Fig. 3). EnsembleAge.Dynamic consistently responded in the expected direction: positive epigenetic age acceleration for stressors and negative age acceleration for rejuvenation treatments (Fig. 3). For instance, the EnsembleAge clock displayed a significant rejuvenation response in murine liver (Z = − 6.96, Fig. 3a) due to caloric restriction (CR). Similarly, the EnsembleAge clock detected substantial stress-induced positive age acceleration in ERCC1-deficient progeria mice (Z = 4.12) mirroring previous results [19].

In several experiments where the original methylation clocks failed to detect the expected effect, the EnsembleAge clocks revealed significant effects. We begin with the example of Huntington’s disease mouse models, where CAG repeat dosage is known to drive neurodegeneration [20]. Despite this established association, the original clocks did not detect any significant epigenetic aging changes in the cerebral cortex or striatum of these mice. In contrast, both EnsembleAge.Dynamic and EnsembleAge.Static identified a clear signal of accelerated aging (dynamic Z = 2.99; static *p* = 0.05; Static.Top *p* = 0.0023; Fig. 3a, b).

On the rejuvenation side, EnsembleAge.Dynamic demonstrated superior sensitivity by detecting the anti-aging effects of rapamycin treatment (Z = − 2.96; Fig. 3a), which the original clocks failed to capture. EnsembleAge.Static still failed to capture a significant (*p* < 0.05) rejuvenation in rapamycin in this experiment (Fig. 3b).

Another noteworthy application of EnsembleAge was in a dataset consisting of murine tail methylation samples collected at postnatal day 20 from 13 different BXD mouse strains [15]. These mice were followed throughout their natural lifespans, which ranged from 1.5 to 2 years, with some receiving a high-fat diet. The original clocks were unable to predict lifespan in this cohort. However, the EnsembleAge.Dynamic and EnsembleAge.Static clocks successfully detected that epigenetic age acceleration in tail samples was significantly associated with earlier mortality (dynamic Z = − 3.04; static *p* = 0.036; Fig. 3d).

Overall, these results highlight the improved sensitivity of EnsembleAge.

### EWAS of ensemble age acceleration

**Fig. 4:**
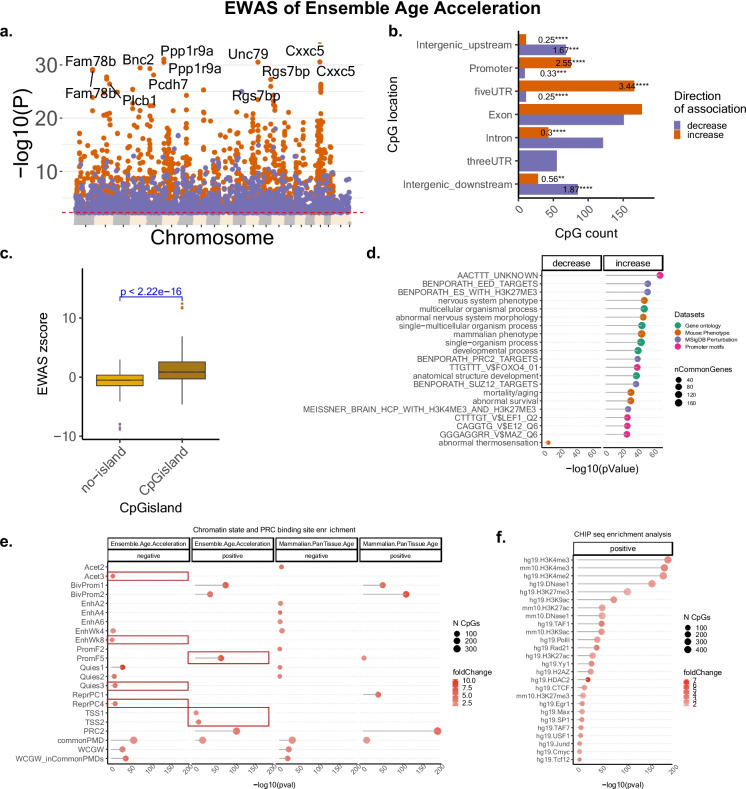
Epigenome-wide association study (EWAS) of dynamic EnsembleAge acceleration. **a** Manhattan plot of EWAS: genome-wide association of EnsembleAge acceleration using 24,898 mammalian CpGs shared between mouse and human. Each point represents a CpG, with the closest mouse gene annotated as the symbol. The *x*-axis shows chromosomal location, while the *y*-axis shows the -log10(*p* value) for each CpG association. Notable genes near significant CpGs include Ppp1r9, and Cxxc5. **b** CpG count by genomic location and enrichment statistics: Distribution of CpGs associated with EnsembleAge acceleration across different genomic regions, including promoters, introns, exons, UTRs, and intergenic regions. Enrichment *p* values and fold changes are reported for each region, showing a significant overrepresentation in 5’UTR regions (*p* < 2.22e−16, fold change = 3.44). **c** CpG island enrichment analysis: Box plot comparing the frequency of CpGs located within CpG islands versus non-island regions. CpGs within islands are significantly enriched for age acceleration signals (*p* < 2.22e−16) based on t-statistics, indicating a strong association with biological age measures. **d** Gene set enrichment analysis of the genes proximal to CpGs associated with EnsembleAge acceleration. We only report enrichment terms that are significant after adjustment for multiple comparisons (hypergeometric FDR < 0.01) and contain at least five significant genes. The top three significant terms per column (EWAS) and enrichment database are shown in the panel. **e** Enrichment of the chromatic states for EWAS hits. The chromatin states are based on the StackHMM, which is the universal chromatic states in humans [22]. The *p* values are calculated by hypergeometric tests of the EWAS results with CpGs that are in each state. The background is limited to the CpGs that could be aligned to the rat genome. The PRC2 state is defined based on the binding motif for any polycomb repressor complex 2 transcriptional factor (EED, SUZ12, EZH2) in human tissues. For comparison, we also included the enrichment results from Pan Mammalian aging results [13], and also highlighted the distinct patterns in EnsembleAge. (f) Enrichment of transcriptional factor motifs and histone marks with DNAm changes associated with Ensemble Age. CpGs are annotated with motifs and histones based on ENCODE ChipSeq data

To characterize EnsembleAge at the single-CpG level, we conducted an EWAS to identify CpG sites linked to epigenetic age acceleration. The EWAS focused on 24,898 CpGs on the mammalian methylation array, which are conserved between mice and humans. Among these, 1291 CpGs demonstrated significant (*p* < 10^−5^) associations with epigenetic age acceleration per dynamic EnsembleAge (Fig. 4a). Notably, CpGs associated with positive epigenetic age acceleration were located in promoter regions and 5’UTRs, indicating that methylation changes in regulatory regions play a key role in positive age acceleration (Fig. 4b). Further, these positively associated CpGs were significantly enriched in CpG islands (Fig. 4c).

In contrast, CpGs associated with negative epigenetic age acceleration were located in intergenic sites. Gene set enrichment [21] of differentially methylated genes highlighted abnormal survival, H3K27me3 and PRC2 marks, and abnormal nervous system and thermosensation, and transcriptional factors such as LEF1, E12 and MAZ (Fig. 4d). Chromatin state enrichment of EnsembleAge revealed several previously unobserved states in age-related EWAS [13] (Fig. 4e highlights the distinct patterns).

We also carried out a chromatin state analysis of the EWAS results using the universal chromatin states [22]. Notably, positive associations with EnsembleAge acceleration were enriched in TSS1, TSS2, and PromF5 regions, while negative associations showed enrichment in Acet3, EnhWk8, Quas3 (stronger in Quas1-2), and ReprPC4 (Fig. 4e). Several of these distinct states (e.g., TSS1, TSS2, and PromF5) are linked to mammalian maximum lifespan [14], further suggesting that Ensemble Age provides a more accurate measure of biological age compared to traditional chronological age analysis.

Key transcription factor motifs showing differential methylation in relation to EnsembleAge included TAF1, Pol II, RAD21, YY1, H2AZ, and HDAC2 (Fig. 4f).

These TFs are well-known regulators of transcription and chromatin structure, and many are implicated in enhancer activity and enhancer-promoter looping either directly or through complex formation: RAD21 and YY1 are directly involved in enhancer-promoter looping. Pol II, TAF1, and H2A.Z are essential for transcriptional activation and can participate in enhancer activity and looping indirectly. HDAC2 fine-tunes enhancer and promoter activity and may influence looping indirectly by altering chromatin accessibility.

Overall, these results suggest that the EnsembleAge clock is associated with transcriptional changes. While we did not analyze transcriptional patterns in this study, previous reports have linked age-associated methylation at several of these loci to changes in gene expression, supporting the potential biological relevance of EnsembleAge markers.

## Discussion

The EnsembleAge clocks are meant to address a major limitation of earlier clocks: lack of sensitivity and inconsistency to assess interventions. By benchmarking clocks against a diverse set of interventions, EnsembleAge improves upon traditional models by leveraging validated perturbation experiments with pro-aging or rejuvenation effects to better reflect biological age—the cumulative effect of genetic, environmental, and lifestyle factors on an organism’s functional capacity. Prior human clocks were based on epidemiological cohorts as opposed to intervention studies. This approach enables a more accurate assessment of health and enhances the ability to detect both pro-aging and rejuvenating effects of interventions. One of the novel properties of EnsembleAge is its ability to exhibit both high sensitivity and specificity when assessing the effects of various interventions, whether they are stress-inducing or rejuvenation-promoting (Fig. 2e). This fine-tuned sensitivity enables the clock to detect even subtle biological changes, making it particularly valuable in studies with limited sample sizes or small-scale interventions where statistical power is often a concern. By integrating multiple regression models and recalibrating predictions based on health outcomes rather than simply chronological progression, EnsembleAge provides a more accurate and robust measure of biological age and the effects of interventions.

In addition to its application as an epigenetic clock, EnsembleAge is supported by a benchmarking framework that enables the ranking of DNA methylation (DNAm) biomarkers. This approach is not limited to clocks but can also be used to assess biomarkers related to healthspan, lifespan, and disease states. By leveraging data from 211 controlled perturbation experiments, this framework ensures the evaluation of the most relevant and robust epigenetic markers across different tissues, species, and experimental conditions. Expanding the range of perturbations and tissues will further improve the predictive power and generalizability of DNAm biomarkers, refining existing models and identifying new molecular indicators of aging and health.

Our chromatin state analysis of the EWAS results for EnsembleAge acceleration identified chromatin states previously linked to age-associated methylation changes across mammalian species, including bivalent promoter regions (BivProm1 and BivProm2) [13]. Strikingly, the analysis also highlighted chromatin states that have been previously associated with maximum mammalian lifespan such as TSS1, TSS2, and PromF5 [14]. Our recent studies have underscored the significance of DNA methylation in highly conserved regions, especially around transcriptional start sites (TSS) and promoter regions, which are constitutively active across species. These regions, including transcriptional start site 1 (TSS1) and promoter flanking (PromF) chromatin states, play a critical role in regulating gene activity essential for survival. Long-living species often exhibit low levels of methylation in these active regions, suggesting an evolved mechanism to sustain high expression levels of crucial survival genes [14]. Notably, these regions did not display DNA methylation changes with chronological age within a species. The enrichment of these states in EnsembleAge suggests that this newly calibrated biological age provides a more accurate measure of longevity compared to traditional chronological age predictors.

The EnsembleAge clock has demonstrated its utility in mouse models and holds potential for broader applications through cross-species analyses. We present HumanMouse versions of EnsembleAge which enable comparative analyses between mice and humans. However, to refine these applications, future studies should focus on expanding MethylGauge with human intervention data. Developing robust human-specific datasets will be essential for benchmarking DNAm biomarkers under controlled conditions and capturing the complexity of genetic, environmental, and lifestyle factors. This will facilitate a deeper understanding of aging and intervention effects while ensuring that cross-species epigenetic markers remain biologically relevant and translationally applicable.

In conclusion, this work establishes a strong foundation for using epigenetic biomarkers like EnsembleAge in preclinical research, and opens the door to future expansion and validation across broader datasets and clinical applications. By gradually building more comprehensive and human-specific datasets, we can refine these biomarkers to capture the full complexity of human biology. Ultimately, this will allow DNAm biomarkers to become powerful tools in precision medicine, enabling more personalized approaches to healthspan interventions and therapeutic treatments.

## Methods

### Mouse and human data

The mouse data used in this study were compiled from multiple publicly available datasets within the Mammalian Methylation Consortium. These datasets, generated by independent research laboratories, include epigenetic data from wild-type C57BL/6 J mice across various ages and tissues. MethylGauge is built on a curated collection of these publicly available datasets, ensuring broad representation of aging-related perturbations. Detailed information regarding Institutional Animal Care and Use Committee (IACUC) approvals, experimental designs, housing conditions, and animal maintenance is reported in the original publications corresponding to each dataset.

### Tail DNA collection and survival follow-up in BXD mice

We utilized DNA methylation data from tail biopsies collected post-weaning from a cohort of BXD recombinant inbred mice, which were part of a larger mouse colony maintained at the University of Tennessee Health Science Center (UTHSC). Following tail sample collection at approximately postnatal day 20, animals were housed under specific pathogen-free conditions and monitored throughout their natural lifespan to record survival outcomes. Mice were assigned to either standard or high-fat diet groups and were periodically weighed, with survival tracked until natural death. Detailed information on animal housing, dietary conditions, and colony management can be found in [15, 23, 24].

### Longitudinal blood sampling and survival tracking in C57BL/6 J mice

We analyzed DNA methylation profiles from longitudinal blood samples collected from a cohort of 81 C57BL/6 J mice (37 females, 44 males) housed at the University of Tübingen. Each mouse was sampled 4 to 5 times over its lifespan, with blood collection beginning at approximately 1.65 years of age and continuing until natural death at up to 2.9 years. Mice were housed under standardized environmental conditions with ad libitum access to food and water, and all procedures were approved by the Institutional Animal Care and Use Committee (IACUC) in accordance with ethical guidelines for animal research. Whole blood was collected at each time point using minimally invasive methods, and genomic DNA was extracted using established protocols.

### Data platform

The DNA methylation data used in this study were generated using either the Mammal40k or Mammal320k BeadChip platforms. To ensure consistency across datasets, the data were merged and restricted to 26,034 mammalian probes that map to both mouse and human genomes [25].

For cross-species analysis, merging mouse and human data required further probe selection to maximize generalizability across platforms. The CpGs were limited to 2252 CpGs that are shared among the Mammal40k, EPIC array, and 450 k array, ensuring compatibility across different methylation platforms.

## Supplementary Information

Below is the link to the electronic supplementary material.

Supplementary Material (XLSX 8.48 MB)

## Data Availability

The MethylGauge dataset is primarily composed of publicly available DNA methylation data. The following GEO (Gene Expression Omnibus) datasets were used in this study: GSE223748, GSE224447, GSE224352, GSE224140, GSE223943, GSE190665, and GSE198652 (Table S1). Detailed experimental conditions, sample processing, and metadata for these datasets can be accessed through their respective GEO entries. Institutional Animal Care and Use Committee (IACUC) details and ethical approvals for publicly available datasets are documented in the cited publications. Genome annotations of these CpGs can be found on Github https://github.com/shorvath/MammalianMethylationConsortium.
